# Tubular Adenoma of the Breast Mimicking Fibroadenoma

**DOI:** 10.7759/cureus.31002

**Published:** 2022-11-02

**Authors:** Mohammed Alfehaid, Abdullah A Hamad Alkharraz, Hamad Alsaeed

**Affiliations:** 1 General Surgery, Qassim University, Unaizah, SAU; 2 General Physician, Ministry of Health, Uniazah, SAU

**Keywords:** beingn breast mass, epithelial neoplasm, breast tumor, tubular adenoma, fibroadenoma

## Abstract

Tubular adenomas of the breast are rare benign epithelial neoplasms and not many cases have been reported. Predominantly, the tumor is described as a palpable, well-circumscribed mass. Most often confused with fibroadenomas clinically and radiologically. Surgical excision is mandatory for diagnosis and to prevent the growth of the mass. Here, we presented a case of these rare tumors. Our case describes a large mass measuring 4.5 × 2.0 × 3.7 cm with tubular adenoma pathology. We elected to document this case to aid in the management of this rare neoplasm. Our aim is to allow physicians to get better identification and treatment for such tumors and improve the outcome for the patients.

## Introduction

Tubular adenoma of the breast is a rare benign epithelial neoplasm, accounting approximately for 0.13% to 1.7% of all benign breast tumors [1]. Predominantly, it occurs in women of childbearing age, rarely before menarche or after menopause [1]. Clinically, tubular adenoma presents with a palpable, well-circumscribed mass [2]. On imaging modalities, these neoplasms have the appearance of noncalcified fibroadenoma. On mammography, the typical appearance is a well-circumscribed nodule, and on ultrasonography, the nodule is described as hypoechoic and well-circumscribed [2]. A precise diagnosis and definitive treatment can only be obtained by histopathological examination after surgical excision [3].

## Case presentation

**Figure 1:**
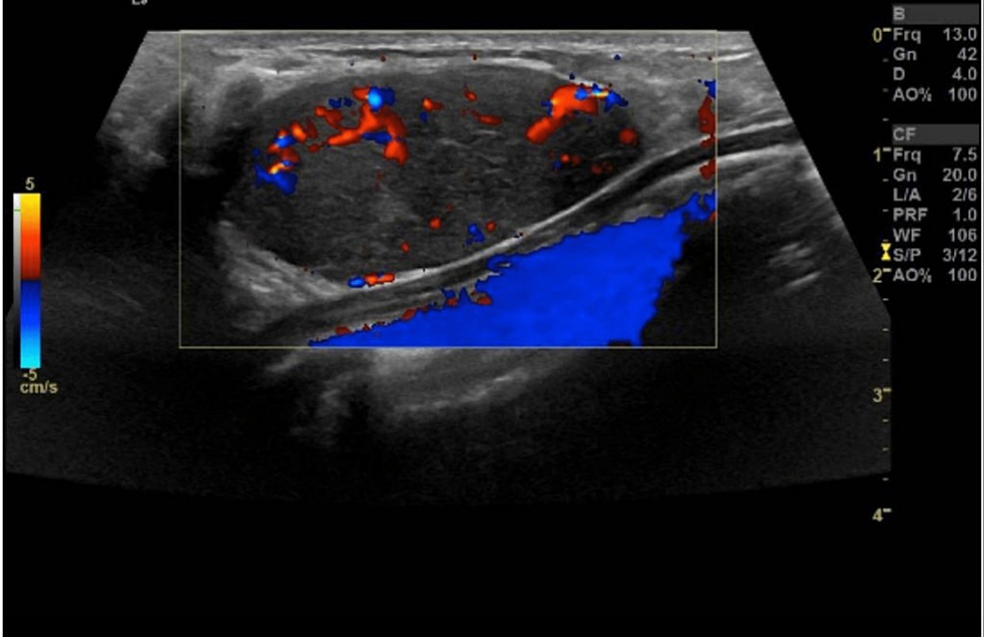
Doppler ultrasonography image of the left breast hypoechoic mass

**Figure 2:**
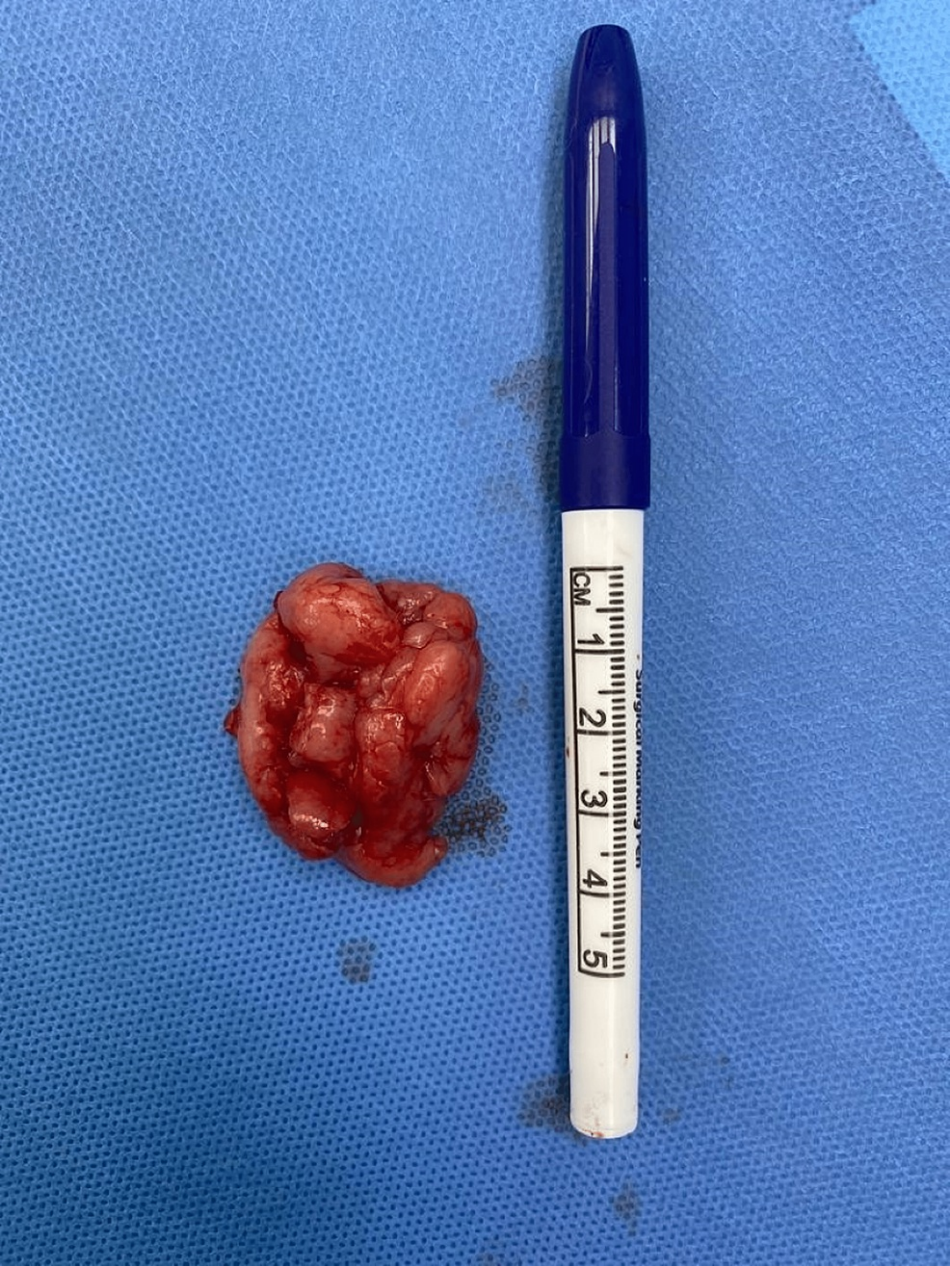
Gross appearance of fibroadenoma

A 19-year-old female, with no significant past medical or surgical history, presented to the general surgery clinic with a complaint of a left breast lump that was noted around six months ago that increased in size with time. Her menstrual cycle was regular with the age of onset of menarche at 12. The patient denied any associated symptoms and reported a family history of breast cancer. On physical examination, a mildly tender, mobile well-circumscribed mass measuring approximately 5 × 4 cm was palpated on the lower inner quadrant of the left breast with no nipple discharge, skin changes, or axillary lymphadenopathy. Ultrasonography demonstrated a large well-defined hypoechoic mass noted at the lower inner quadrant at the 6-9 o'clock position of the left breast measuring 4.5 × 2.0 × 3.7 cm concerning fibroadenoma. No internal calcification, cystic changes, or abnormal axillary lymphadenopathy were noted. An oval/elliptical lymph node with the usual benign configuration of fatty hilum and hilar vascularity is noted in the left axillary region measuring 1.3 × 0.5 cm. On color Doppler, there was central and peripheral vascularity (Figure 1). Surgical excision was performed (Figure 2). The histopathology report showed tan and firm tissue fragments measuring in aggregate 4.0 × 3.0 × 2.0 cm, suggestive of tubular adenoma with no malignant features. The patient was discharged on the same day and visited the clinic after one week for follow-up without any complaints.

## Discussion

Breast tubular adenomas are rare epithelial tumors that are mostly benign and occur in females of childbearing age [4,5]. It is typically presented as palpable, mobile, non-tender mosses with skin or nipple change [6]. On imaging, tubular adenomas usually look like fibroadenomas, as in they look noncalcified on a mammogram and well-circumscribed hypoechoic on ultrasound [2]. A definitive diagnosis of breast tubular adenoma can only be reached by histopathology due to their characteristic appearance of proliferating round and uniform tubules lined by regular epithelial cells surrounded by myoepithelial cells, packed in a small amount of stroma [7]. As in any other growing breast mass, the management of breast tubular adenoma is started by history, physical exam, and imaging whether ultrasound or mammogram. Due to their benign nature and the patients mostly complaining about the increasing size, they are managed with surgical excision like a fibroadenoma. No follow-up had been reported in the literature except for one case with an 18-month follow-up with no recurrence [1]. Malignancy can be a differential diagnosis of tubular adenoma. In the literature, it has been reported in very few cases. Saimura et al. discovered histopathological findings of ductal carcinoma in situ within tubular adenoma tissue and Domoto et al. described co-localization of a tubular adenoma with invasive ductal carcinoma; however, they found that malignant transformation cannot be ruled out [8,9]. In our case, the patient presented with benign features as in fibroadenoma and was described in the histopathology report as a 4.0 × 3.0 × .2.0 cm tubular adenoma with no malignant features. Very few cases were published in the literature. A case report was released in 2019 on a young female who presented with bilateral breast masses for six months, 2 × 3 cm on the right and left breast with no associated symptoms. The patient had a significant family history of breast cancer. Ultrasound findings revealed both well-defined hypoechoic masses with no malignant features. surgical excision was done, and a histopathology report showed a tubular adenoma with no evidence of malignancy [10]. Furthermore, two cases were published in 2018. The first case was a young female with no significant past medical or surgical history who presented with a right breast mass of 10 × 9.5 × 4 cm with no other symptoms. A core needle biopsy was done which resulted in a tubular adenoma, and a histopathological report confirmed it. Their second case was a young female that complained of a painful and tender left breast mass, the size of approximately 2 cm, and a history of bloody nipple discharge. The patient had a positive family history of breast cancer. After surgical excision revealed mixed features of tubular adenoma and fibroadenoma. Ultrasound demonstrated a well-circumscribed hypoechoic mass with posterior enhancement. After surgical excision histopathology specimen revealed mixed features of tubular adenoma and fibroadenoma with no malignancy [6].

## Conclusions

Tubular adenoma of the breast is a rare benign neoplasm commonly confused with fibroadenoma due to its similarity in presentation and pre-operative evaluation. Surgical excision and histopathological examination are necessary to obtain a definitive diagnosis. However, this type of adenoma is not common and rarely malignant hence, it should be always considered a differential diagnosis for benign breast lump.
